# Profile of Caregiving Activities and Association With Physical Health Among Dementia Spousal Caregivers

**DOI:** 10.1093/geroni/igae017

**Published:** 2024-02-15

**Authors:** Jinmyoung Cho, Laura P Sands, Alan B Stevens, Heather G Allore, Molly J Horstman

**Affiliations:** Department of Family and Community Medicine, Saint Louis University School of Medicine, Saint Louis, Missouri, USA; Center for Applied Health Research, Baylor Scott & White Research Institute, Temple, Texas, USA; Center for Gerontology, Virginia Tech, Blacksburg, Virginia, USA; Center for Applied Health Research, Baylor Scott & White Research Institute, Temple, Texas, USA; Department of Medicine, Baylor College of Medicine, Houston, Texas, USA; Department of Biostatistics, School of Public Health, Yale University, New Haven, Connecticut, USA; Section of Geriatrics, Department of Internal Medicine, School of Medicine, Yale University, New Haven, Connecticut, USA; Department of Medicine, Baylor College of Medicine, Houston, Texas, USA; Center for Innovations in Quality, Effectiveness, and Safety, Michael E. DeBakey VA Medical Center, Houston, Texas, USA

**Keywords:** Cluster analysis, Intensity of caregiving, Multidimensional health indicators

## Abstract

**Background and Objectives:**

This study aims to identify patterns of caregiving intensity and assess associations between caregiving intensity and multidimensional physical health indicators and health behaviors among spousal caregivers of persons with Alzheimer’s disease and related dementia.

**Research Design and Methods:**

Using data from 152 spousal caregivers aged 65 and older, the intensity of their caregiving experience was measured as the number and frequency of health- and medical-related helping activities for their care recipient. Multidimensional health indicators included self-reported fatigue, sleep disturbance, physical functioning, pain interference, general health, and the number of chronic conditions from the electronic health records. Self-reported health promotion behaviors were assessed as health responsibility, physical activity, nutrition, interpersonal relations, and stress management.

**Results:**

Two distinct caregiving intensity patterns, high-intensity (37.5%) and low-intensity (62.5%) caregiving, were identified with cluster analysis. Caregivers in the high-intensity caregiving cluster reported feeling more tired (*t* = 2.25, *p* < .05), experiencing more sleep disturbance (*t* = 3.06, *p* < .01), and performing less physical activity (*t* = 2.05, *p* < .05) compared with caregivers in the low-intensity group.

**Discussion and Implications:**

Future studies are needed to develop effective interventions to address caregiving intensity and its consequences on the health of spousal caregivers of persons with dementia.

**Translational Significance:** The demands of caring for a person with Alzheimer’s disease and related dementia (ADRD) can limit the spousal caregiver’s time and efforts to take care of themself. Little is known about the impact of caregiving intensity on spousal caregivers’ physical health outcomes. We identified two clusters of caregiving intensity (high vs low) and found that high-intensity caregiving was associated with higher levels of fatigue and sleep disturbance, and lower levels of physical activity. Linking caregiving intensity to specific health risks emphasizes the importance of developing strategies that reduce caregiving intensity among spousal caregivers of persons with ADRD.

Family caregiving is a valued tradition in society and has become an essential element of the U.S. healthcare system. More than 80% of the long-term care provided to older adults in the United States comes from family members or other unpaid helpers (Friedman et al., 2015). The number of individuals engaged in family caregiving for people living with dementia will increase dramatically in the coming decades. Thus, understanding the impact of family caregiving on the health risks and outcomes of those providing care is essential for developing interventions and policies to reduce health burdens associated with caregiving. Taking care of family members with Alzheimer’s disease and related dementia (ADRD) is not without risk to family caregivers. Studies have demonstrated that caring for family members living with ADRD is associated with significant levels of daily burden, increased stress and depression, and reduced quality of life for caregivers (Pinquart et al., 2003). As the prevalence of age-related diseases increases in older adults, spouses of persons with ADRD are at a higher risk for health problems than spouses of persons without ADRD (Kolanowski et al., 2004). Caregivers over age 65 have a greater prevalence of chronic conditions, including hypertension, arthritis, and heart disease, than noncaregivers over age 65 (Wang et al., 2014). Importantly, health ratings of “fair” or “poor” are associated with higher healthcare utilization (Schulz & Cook, 2011), including Emergency Department utilization.

The Stress Process Model guides the hypotheses and methods of this study (Pearlin et al., 1990). This model is commonly used in studies of dementia caregiving because it explains how primary stressors (caregiving experiences) influence caregivers’ outcomes (caregiver health). Pearlin (2010) also emphasized that the linked lives and role set, hallmarks of the life course framework, are useful in the study of the stress process. Under some conditions, the stressors that one person experiences can become primary stressors for their partners (Pearlin, 2010). In the context of dementia caregiving, persons who provide care for their spouse with dementia often experience stress and burden associated with the daily routine of providing dementia care. Spousal caregivers may be at a greater risk of adverse health outcomes than non-spousal caregivers due to the historical (decades) and emotional aspects associated with a spousal relationship (i.e., linked lives and role expectations). Further, the age of caregivers is a significant factor in caregiver outcomes (Polenick et al., 2020; Rosland et al., 2010). Age-related physical and mental health changes (mostly declines) are commonly observed in older adults, and spousal caregivers are typically older than non-spousal caregivers, indicating that many spousal caregivers are vulnerable to the onset of chronic health conditions.

Little is known about how the intensity of caregiving activities varies among dementia caregivers and how caregiving intensity affects caregivers’ health and health behaviors, although prior studies have shown that the heavy daily demands of caregiving are associated with increases in caregivers’ stress and burden. Compared with other types of caregivers (e.g., children, child-in-laws), spousal caregivers’ personal, physical, and psychological needs often take a back seat to the care recipients’ needs (Bakas et al., 2002; Clark et al., 2008; Edelman et al., 2006; Hileman et al., 1992; Tommis et al., 2009; Wenger et al., 2002). Spousal caregivers of persons with ADRD have reported that they neglected their own healthcare because they devoted their time to caring for the spouse with ADRD (Wright, 1994). Thus, there is a need to better understand the impact of caregiving intensity on spousal caregivers’ health outcomes.

Prior research on caregiving intensity has relied on either counting the number of instrumental activities of daily living (IADLs) caregivers assist with or the number of hours caregivers spend helping with IADL tasks (Chen et al., 2020; Fredman et al., 2019; Kolodziej et al., 2022; Meyer et al., 2022). For example, in a 2020 U.S. study, caregiving intensity was assessed by the number of hours spent caregiving per week and the number of IADL activities the caregiver provided help with to classify the level of caregiving burden as low, medium, or high (National Alliance for Caregiving, 2020). However, assessing only the number of hours of caregiving focusing on IADL activities neglects the many types of non-IADL caregiving activities that the spouses provide for their care recipient. Further, because most spousal caregivers of persons with ADRD provide more hours of caregiving than non-spousal caregivers, specifying the measure of caregiving intensity by incorporating the number and frequency of more than IADL activities for persons with ADRD could improve our understanding of the caregiving experience faced by spousal caregivers.

The purpose of this study is to develop a better understanding of the association between caregiving intensity and spousal caregivers’ health experiences. This study aims to identify the caregiving intensity using 23 caregiving activities and to assess the association of caregiving intensity on multidimensional physical health indicators and health behaviors among spousal caregivers for persons with ADRD. Findings may inform risk identification and the need for interventions to reduce poor health outcomes associated with caregiving intensity.

## Method

### Recruitment, Screening, Inclusion and Exclusion Criteria, and Enrollment

The current study included data from 152 spousal caregivers who provided help for their spouse with ADRD. We used two recruitment strategies. First, the participants were recruited through physician referrals within an integrated healthcare system located in a south-central state of the United States. This was accomplished by the research team generating a list of patients with a dementia diagnosis (ICD-9/10 codes) in patients’ health records and providing the list to their physicians with study information. Physicians could then introduce the study to the patients and/or caregivers who were a good fit for the study. Research staff followed up with the patients and/or caregivers. Second, the research team partnered with other research projects relevant to patients with ADRD that used the same physician referral process. Patients who were not involved in another research study and whose caregivers agreed to be contacted for relevant research were referred to this study.

The research team determined the eligibility of caregivers who were interested in participating in the current study. The inclusion criteria were that they were 65 years and older, English speakers, the spouse of the care recipient with dementia, they provided at least 8 hr of care per week for their spousal care recipient, and that both the care recipient and caregiver were patients at Baylor Scott & White Health (BSWH) to identify persons with dementia and to assess spousal caregiver’s chronic conditions through their health records. To corroborate ADRD diagnoses from the patient records, the spousal caregiver was asked to complete the AD8, which includes an assessment of the care recipients’ memory, orientation, executive function, and interest in activities with a yes/no scoring system (Galvin et al., 2005). An AD8 score of two or greater was required for this study.

**Figure 1.**
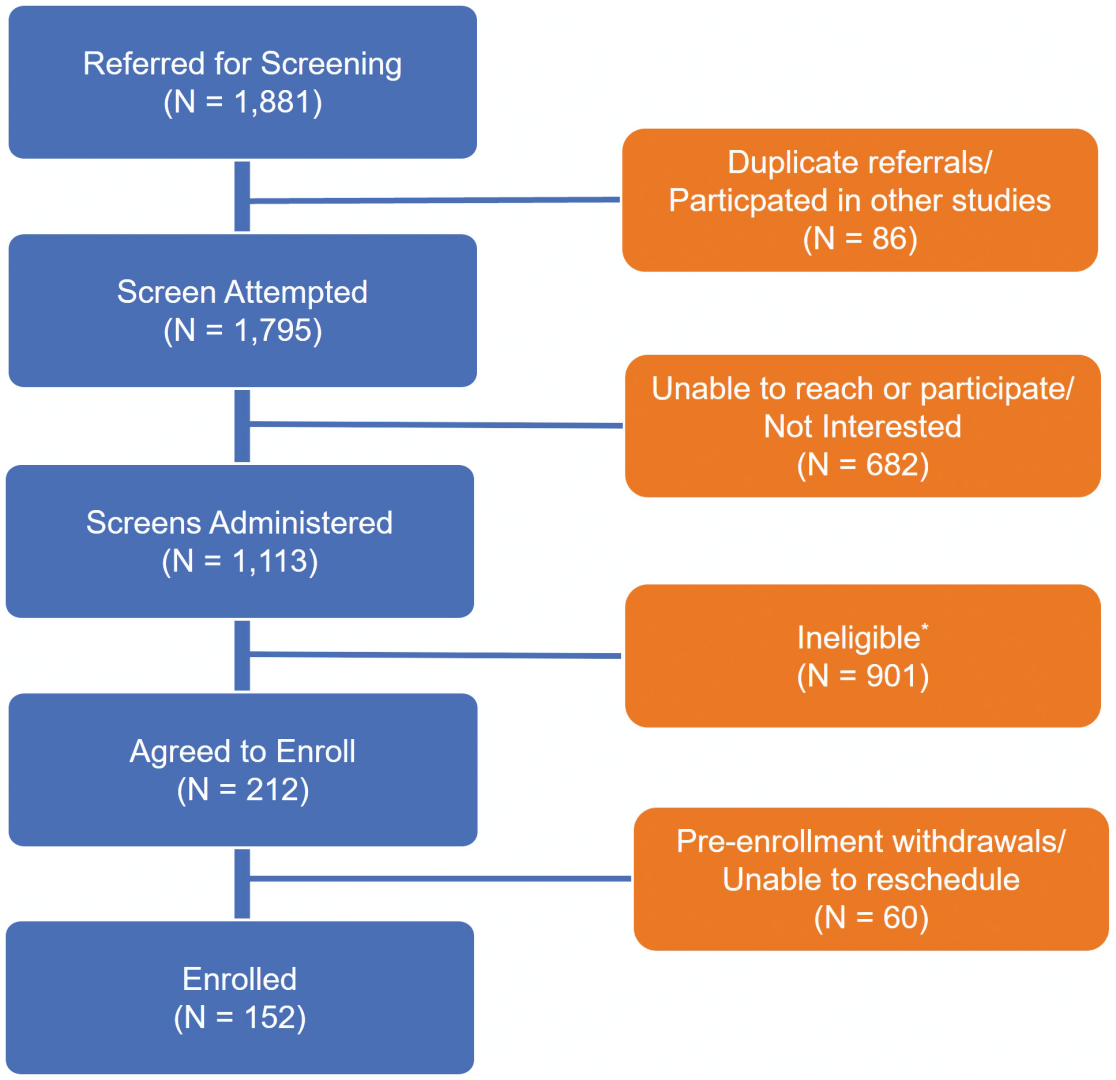
Study participant recruitment process. *Ineligible includes: caregiver is not a spouse; death of caregiver; caregiver is not a Baylor Scott & White Health patient; care recipient’s AD 8 score is lower than 2; caregiver is younger than 65 years; providing less than 8 hr of care weekly; or caregiver is a non-English speaker.

As shown in Figure 1, a total of 1,881 patients were referred to the research team. A total of 1,113 patients and their family members were assessed for eligibility. Over 900 were excluded because primary caregivers were not a spouse, not a BSWH patient, younger than 65 years old, not an English speaker, and provided less than 8 hr of care per week. The current study includes 152 spousal caregivers who completed survey calls from October 2021 to December 2022.

### Data Collection

After confirming eligibility, a study packet was mailed or emailed to caregivers interested in participating in the study. The study packet included an informed consent form, informed consent form instructions, a cover letter, survey questions, and a return self-addressed envelope. For caregivers who were interested in the $20 gift card for study participation, a blank W9 form, W9 form instructions, and a gift card information document were also included in the study packet. Depending on the caregiver’s preference, the caregiver was directed to the project REDCap website and provided a link to access the informed consent document. Alternatively, the caregiver was provided a paper copy of the consent form in the study packet. Once the research team obtained the caregivers’ consent to participate in the study, an online or phone call interview was completed. All data collection systems and services were captured and stored using REDCap, the Clinical and Translational Science Awards-supported data management tool (https://www.project-redcap.org/). A local hospital’s Institutional Review Board approved this study.

### Measures

#### Caregiving activities

Caregiver’s help with 23 health- and medical-related tasks was categorized into five domains (Riffin et al., 2019; Wolff et al., 2016): activities of daily living (ADL)-related activities (5 items), IADL-related activities (5 items), mobility activities (3 items), health system logistics (5 items), and health management (5 items). For help with ADL-related activities, caregivers were asked about how often they assisted their care recipients with: (a) eating, (b) showering/bathing, (c) dressing/grooming, (d) using the toilet, and (e) getting in and out of bed. The IADL-related activities included (a) laundry or cleaning, (b) making hot meals, (c) shopping for groceries or personal items, (d) getting around the house, and (e) driving. For help with mobility activities, caregivers were asked how often they assisted with (a) lifting him/her from a seated or lying position, (b) letting him/her lean on them to support his/her weight, and (c) holding him/her steady while he/she walked or stood. The health system logistics domain included making appointments, ordering medicines, handling insurance issues, keeping track of medications, and speaking with the patient’s medical provider. Lastly, the health management domain consisted of assisting five tasks: diet, foot care, skin care, exercise, and dental care. Each response was scored as follows: 0 = never, 1 = rarely, 2 = sometime, 3 = most of the time, and 4 = all the time. A summary score of participant’s responses for each domain was used to count the number and frequency of health- and medical-related helping activities for his/her care recipient. Possible ranges for each domain were from 0 to 20 for ADL-related activities, IADL-related activities, health system logistics, and health management. The possible summary score for mobility activities ranged from 0 to 12. Cronbach’s alphas in this study were 0.858 for ADL-related activities, 0.682 for IADL-related activities, 0.885 for mobility activities, 0.837 for health system logistics, and 0.694 for health management, respectively.

#### Physical health indicators

Two aspects of caregivers’ physical health were self-reported indicators and diagnoses from the caregiver’s electronic health records (EHR). The Patient-Reported Outcomes Measurement Information System (PROMIS®) was used to assess self-reported physical health. The PROMIS® is a set of person-centered measures that evaluate physical, mental, and social health and has been used in the general population and with groups of patients with chronic conditions. Self-reported physical health includes fatigue (13 items), pain intensity (3 items), pain interference (6 items), physical functioning (10 items), sleep disturbance (8 items), overall health (2 items), and self-efficacy (4 items). All responses on a 1 (not at all) to 5 (very much) Likert scale were summed. The summary scores of each domain at the participant level were converted to T-scores with a mean of 50 and a standardized deviation (*SD*) of 10. A T-score of 50 on each domain is the average for the U.S. general population, and the 10 *SD* represents 1 *SD* from the general population mean (Kroenke et al., 2018). Diagnoses extracted from caregivers’ EHR were used to count the number of diagnosed chronic conditions. A total of 30 chronic conditions were classified according to CMS ICD-9/10 codes (Centers for Medicare & Medicaid Services, 2017), such as diabetes, hypertension, and acquired hypothyroidism (ICD-9/10 codes for each condition available upon request). The range of possible chronic conditions was 0–30.

#### Health promotion behaviors

Health promotion behaviors were assessed using five subscales of the Health Promoting Lifestyle Profile-II: health responsibility, physical activity, nutrition, interpersonal relations, and stress management (Walker et al., 1987, 1988, 1990). Participants were asked to rate the frequency of their practicing these 43 behaviors. A 4-point Likert scale from 1 (never) to 4 (always) was used in each item. The sum of the scores of items in each subscale was used to represent each health promotion behavior; higher scores indicate a higher level of health promotion behaviors. Possible ranges for each subscale were from 9 to 36 for health responsibility, nutrition, and interpersonal relations; and from 8 to 32 for physical activity and stress management. The Cronbach’s alpha of each subscale is 0.734 for health responsibility, 0.800 for physical activity, 0.726 for nutrition, 0.764 for interpersonal relations, and .730 for stress management, respectively.

### Statistical Analyses

Descriptive statistics were calculated for participant characteristics collected at enrollment. To characterize the intensity of the caregiving experience, we performed both hierarchical and *k*-means cluster analysis using variables on the number and frequency of helping with five domains of activities including: ADL, IADL, health management, health system logistics, and mobility. In data preprocessing, these variables were normalized using *z*-scores. We first performed hierarchical clustering using a Euclidean distance function with the proximity between groups of variables measured using Ward’s method. A dendrogram was plotted to depict the similarity in relationships among all caregivers. After determining the optimal number of clusters, *g*, in hierarchical clustering, we performed *k*-means clustering specifying *g* number of clusters (Husson et al., 2014). The means of the standardized variables were used as the initial seeds of the *k*-means. Prior specification of the number of clusters and initial centralized procedure are more powerful and reliable than hierarchical procedures. Furthermore, this procedure helped improve the assignment of participants to clusters and obtain the best solution (Mouriz-Corbelle et al., 2021). We also compared caregiver’s characteristics associated with caregiving intensity. Then, we conducted *t*-tests to assess which physical health indicators and health behaviors differed for the two clusters. Analyses were conducted using IBM SPSS Statistics version 29.0.

## Results

### Characteristics of the Participants

Table 1 shows the characteristics of 152 spousal caregiver participants who completed study calls. The average age was 77.2 years (*SD* = 6.47). The majority were women (63.2%), white/Caucasian (94.1%), and non-Hispanic/Latino (95.4%). More than 85% were retirees. Over half of the participants completed college education and above, and the annual household income was less than $100K for over 70% of the caregiver participants. Half of caregiver participants reported that their care recipient was diagnosed with either Alzheimer’s disease (41.9%) or dementia (48.4%). The rest of the diagnoses include vascular dementia or transient ischemic attacks, Parkinson’s disease, Lewy body disease, frontotemporal disease, and mild cognitive impairment. Most caregivers stated that they provide up to 8 hr daily (75.0%) and have provided care for at least 2 years (81.6%).

**Table 1.** Participant’s Characteristics (*N* = 152)

Participant’s characteristics	All participants	High-intensity (*n* = 57)	Low-intensity (*n* = 95)
	Mean [*SD*] or *N* (%)	Mean [*SD*] or *N* (%)	Mean [*SD*] or *N* (%)
Age	77.2 [6.47]	76.5 [6.99]	77.6 [6.13]
Sex			
Female	96 (63.2%)	37 (64.9%)	59 (62.1%)
Male	56 (36.8%)	20 (35.1%)	36 (37.9%)
Race			
White/Caucasian	143 (94.1%)	52 (91.2%)	91 (95.8%)
Other^a^	9 (5.9%)	5 (8.8%)	4 (4.2%)
Ethnicity			
Latino	7 (4.6%)	3 (5.3%)	4 (4.2%)
Employment status			
Retired	130 (85.5%)	51 (89.5%)	79 (83.2%)
Full-time/part-time/unpaid working^b^	22 (14.5%)	6 (10.5%)	16 (16.8%)
Education			
Less than college graduate	75 (49.4%)	27 (47.4%)	48 (50.5%)
College graduate or above	77 (50.6%)	30 (52.6%)	47 (49.5%)
Annual household income*			
Less than $50K	41 (27.0%)	22 (40.7%)	19 (22.4%)
$50K to 100K	70 (46.1%)	27 (50.0%)	43 (50.6%)
$100K+	28 (18.4%)	5 (9.3%)	23 (27.1%)
Declined	13 (8.6%)	—	—
Diagnosis			
Alzheimer’s disease	48 (31.6%)	22 (38.6%)	26 (27.4%)
Dementia	62 (40.8%)	23 (40.4%)	39 (41.1%)
Vascular dementia or TIAs	6 (3.9%)	2 (3.5%)	4 (4.2%)
Parkinson’s disease	6 (3.9%)	2 (3.5%)	4 (4.2%)
Lewy body disease	5 (3.3%)	3 (5.3%)	2 (2.1%)
Frontotemporal disease	10 (6.6%)	3 (5.3%)	7 (7.4%)
Mild cognitive impairment	7 (4.6%)	1 (1.8%)	6 (6.3%)
Other/unsure	8 (5.3%)	1 (1.8%)	7 (7.4%)
Daily hours of providing care***			
Up to 4 hr	61 (40.1%)	3 (5.4%)	58 (61.1%)
5–8 hr	53 (34.9%)	28 (50.0%)	25 (26.3%)
9–15 hr	15 (9.9%)	11 (19.6%)	4 (4.2%)
16+ hr	22 (14.5%)	14 (25.0%)	8 (8.4%)
Duration of providing care			
6 months to 2 years	28 (18.4%)	10 (17.5%)	18 (18.9%)
2–5 years	70 (46.1%)	23 (40.4%)	47 (49.5%)
5+ years	54 (35.5%)	24 (42.1%)	30 (31.6%)

Notes: TIAs = transient ischemic attacks.

^a^Other includes Black/African American, American Indian/Alaskan Native, Asian, unknown.

^b^Unpaid working includes volunteer activities and homemaker.

*p < .05;

***p < .001.

### Characteristics of Intensity of Caregiving Experience

Cluster analysis revealed two clusters of caregiving intensity: high-intensity (37.5%) and low-intensity (62.5%) caregiving. The mean scores (SDs) of the five domains of caregiving activities are presented in Table 2. Compared with the low-intensity caregiving cluster, the high-intensity caregiving cluster had significantly higher values in all five domains: ADL-related activities = 11.28 (vs. 2.01 for low-intensity cluster; *t*(150) = 15.25, *p* < .001), IADL-related activities = 17.70 (vs. 12.27 for low-intensity cluster; *t* = 10.11, *p* < .001), mobility activities = 6.02 (vs. 1.76 for low-intensity cluster; *t* = 8.72, *p* < .001), health system logistics = 19.54 (vs. 16.48 for low-intensity cluster; *t* = 4.69, *p* < .001), and health management = 13.31 (vs. 4.71 for low-intensity cluster; *t* = 13.66, *p* < .001). In addition, Table 1 shows significant differences between the two intensity groups in the daily hours of care provided (χ^2^ = 49.24, *p* < .001). Notably, 44.6% of high-intensity caregivers reported more than 9 hr of daily caregiving compared with only 12.6% of low-intensity caregivers.

**Table 2.** Comparisons in Frequencies of Caregiving Activities by Caregiving Intensity

Caregiving activities	High intensity (*n* = 57)	Low intensity (*n* = 95)	*t* (150)	*p*
ADL-related activities (0–20)	11.28 (5.04)	2.01 (2.43)	15.25	<.001
IADL-related activities (0–20)	17.70 (1.99)	12.27 (3.75)	10.11	<.001
Mobility activities (0–12)	6.02 (3.56)	1.76 (0.58)	8.72	<.001
Health system logistics (0–20)	19.54 (1.75)	16.48 (4.75)	4.69	<.001
Health management (0–20)	13.31 (0.75)	4.71 (3.44)	13.66	<.001

*Note*: ADL = activities of daily living; IADL = instrumental activities of daily living. Mean and standard deviation values for each of the analyses are shown. Results of *t*-test assuming equal variance compare the parameter estimates between the two clusters. High scores indicate that caregivers help activities more often.

### Caregiver Characteristics by Caregiving Intensity

In addition to calculating caregiver’s characteristics overall (Table 1), we compared them by caregiving intensity. There were no significant differences between high and low caregiving intensity clusters in age, sex, race, ethnicity, employment status, education, and types of dementia. However, there was a significant difference in annual household income (χ^2^ = 8.98, *p* < .05). Over a quarter of caregivers in the low-intensity cluster (27.1%) reported their annual household income is higher than $100K compared with 9.3% of those in the high-intensity cluster.

### Physical Health Indicators and Health Promotion Behaviors by Caregiving Intensity

Table 3 summarizes the comparison of physical health indicators and health promotion behaviors by caregiving intensity. Caregivers from the high-intensity caregiving cluster reported feeling more tired (*t *= 2.16, *p < *.05), perceiving more sleep disturbance (*t *= 2.94, *p < *.01), and performing less physical activity (*t *= 2.05, *p < *.05), compared with caregivers from low-intensity caregiving cluster. Other health indicators and health behaviors did not show significant differences between the two clusters.

**Table 3.** Comparisons in Physical Health Indicators and Health Behaviors by Caregiving Intensity

Health indicators	High intensity (*n* = 57)	Low intensity (*n* = 95)	*t* (150)	*p*
PROMIS Health indicators (self-report)				
Fatigue	53.61 (8.78)	50.60 (8.03)	2.16	.032
Sleep disturbance	51.70 (10.40)	47.04 (8.86)	2.94	.004
Pain interference	51.65 (8.97)	49.53 (9.16)	1.39	.165
Pain intensity	43.96 (9.46)	41.93 (10.03)	1.23	.219
Physical functioning	45.47 (9.52)	46.44 (9.56)	0.97	.545
General health	47.59 (7.37)	48.75 (8.44)	0.86	.393
Self-efficacy	51.83 (10.06)	50.97 (9.20)	1.10	.274
Health promotion behaviors (self-report)				
Health responsibility	2.59 (0.52)	2.54 (0.59)	.61	.546
Physical activity	1.91 (0.75)	2.16 (0.70)	2.05	.043
Stress management	2.59 (0.55)	2.76 (0.63)	1.68	.095
Nutrition	2.74 (0.55)	2.78 (0.58)	.36	.717
Interpersonal relations	2.91 (0.50)	2.86 (0.57)	.54	.592
Number of chronic condition diagnoses from EHR	6.12 (2.84)	5.80(3.01)	0.66	.508

*Note*: PROMIS = The Patient-Reported Outcomes Measurement Information System; EHR = electronic health records. Mean and standard deviation values for each of the analyses are shown. Results of *t*-test assuming unequal variance compare the parameter estimates between the two clusters.

## Discussion

This study highlights the association between caregiving intensity and multidimensional health indicators and self-reported health behaviors among spousal caregivers of persons living with ADRD. We identified two clusters of caregiving intensity using 23 caregiving activities in five domains. A major finding of this study is that there were significant differences in health indicators and health behaviors for those who experienced high- versus low-intensity caregiving. The high-intensity caregiving cluster showed significantly higher levels of fatigue and sleep disturbance, and lower levels of physical activity compared with the low-intensity caregiving cluster.

Our study is unique in its measurement of caregiving intensity. The measure of caregiving intensity was based on the frequency of assisting with 23 activities across multiple dimensions of caregiving. This approach provides a holistic assessment of caregiving activities, as compared with prior studies that simply assessed how many hours were spent providing caregiving or focused only on I/ADL activities. Estimating the number of hours spent on caregiving can be problematic for caregivers who typically do not keep track of the amount of time they spend assisting with tasks for care recipients (Fredman et al., 2019). Further, spousal caregivers may have difficulty assessing the number of hours spent supporting their spouse with I/ADL activities because many of these activities (e.g., preparing meals, laundry, etc.) are part of their everyday lives. In contrast, asking spousal caregivers to report the number and frequency of health- and medical-related activities they provide for their care recipient with ADRD more explicitly captures tasks associated with caring for someone with ADRD. Our approach is in contrast to prior studies that based intensity on either hours of care provided or counts of tasks. The findings of this study validated our approach to measuring caregiving intensity by showing that the frequency of caregiving across five domains of caregiving activities was significantly higher in the high-intensity group (Table 2). Furthermore, Table 1 provides additional evidence of the construct validity of our classification by showing that 61% of those in the low-intensity group provided four or fewer hours of care, compared with 5% of those in the high-intensity group (*χ*^2^ = 49.24, *p* < .001).

The findings of this study are also significant in that we examined the multiple aspects of the physical health of spousal caregivers for people with ADRD. To the best of our knowledge, this is the first study to examine various dimensions of physical health in the context of dementia caregiving intensity. Strengthening the evidence from earlier studies (Rabinowitz et al., 2007; Waligora et al., 2018), we observed a possible negative effect on the physical health of caregivers based on the intensity of the caregiving they provided. Specifically, spousal caregivers with high-intensity caregiving reported greater lack of sleep, fatigue, and reduction in physical activities compared with their counterparts with low-intensity caregiving. This result indicates that spousal caregivers for care recipients with dementia are in need of interventions that emphasize the importance of practicing self-care and self-management to promote optimal health outcomes for spousal caregivers (Waligora et al., 2018).

One important feature of our method for assessing physical health is that we included an objective measure to assess the health status of spousal caregivers: the number of chronic conditions. The number of chronic conditions was assessed by counting classifications of CMS chronic conditions from ICD-9/10 codes from the caregiver’s EHR. Compared with objective measures, self-reports on participant’s chronic conditions are subject to potential recall biases, which could compromise the accuracy of their self-reported health conditions. Assessing the presence of multiple chronic conditions is important for identifying patients in need of extra coordination of medical care or support (Suls et al., 2022). Furthermore, using scales from the PROMIS® allowed the comparison of each domain to the general population. Surprisingly, the T-scores of all physical health domains (ranges from 42.7 to 51.7) were within a normal range for the general population (Cella et al., 2010; Nagaraja et al., 2018).

Several mechanisms may explain the results on the multiple aspects of physical health. First, we did not find significant differences between the two caregiving intensity clusters in the number of chronic conditions. This may be because this study compares two groups of spousal caregivers aged 65 years and older. Most Americans over 65 years of age have at least one chronic condition, and 60% have at least two chronic conditions (National Center for Chronic Disease Prevention and Health Promotion, 2019). The average number of chronic conditions among spousal caregivers in our study was 5.92, and over 95% of them had at least two chronic conditions listed in the EHR regardless of caregiving intensity. So, a significant or large difference in chronic conditions between the clusters would not be expected, as has been found in prior studies that compared caregivers with noncaregivers. Second, the allostatic load theory may explain this finding (Rabey & Moloney, 2022; Read & Grundy, 2014). The onset of stress related to chronic conditions may have developed over time. Most caregivers in both clusters had been providing care to their care recipient for at least 2 years. Although they might not notice gradual changes in their health, stress-related biological burden continuously accumulates as dementia patients’ needs increase over time. The increasing care demands might decrease their awareness of their health needs as the intensity of caregiving activities increases.

In assessing caregiving characteristics, we found significant differences in annual household incomes between high- and low-intensity clusters. Caregivers in the low-intensity cluster reported higher incomes compared with caregivers in the high-intensity cluster. This finding indicates that high-income caregivers may have greater access to tangible resources that are useful in meeting the daily care needs of a spouse with dementia. Although we did not have an a priori hypothesis that income would differ between caregiving intensity groups, this finding suggests that greater access to care supports that could mitigate the level of intensity caregiving and thus reduce the negative health consequences experienced by spousal caregivers. Future research is needed to understand better the link between financial resources, level of caregiving intensity, and health outcomes of spousal caregivers.

This study also has a methodological significance in recruiting study participants within a healthcare system. In real-world settings, many researchers struggle with identifying caregivers for people with ADRD (Joshi et al., 2023). In this study, the research team identified patients with ADRD from the EHR and partnered with primary care providers within an integrated healthcare system. This recruitment strategy enabled the research team to identify and contact nearly 1,800 patients with ADRD and their family members. This was an effective and collaborative strategy to recruit caregivers for a patient with dementia within a healthcare system.

We acknowledge several limitations of this research. First, the study participants were recruited from a healthcare system located in only one geographic area of the United States. Caregivers located in different regions may present different findings. Furthermore, the majority of study participants identified themselves as a non-Hispanic White. Caregivers from diverse racial-ethnic groups may report different levels of physical health status and health behaviors. As shown in Table 1, over 25% of participants from the low-intensity caregiving cluster reported $100K+ in annual household income. Study participants were recruited from a large integrated healthcare system that provides a full range of medical services to patient populations in rural, suburban, and urban communities; however, individuals with low-income may have faced barriers to accessing care services, which may limit the research to recruit low-income caregivers into the study. Future research should consider the association between financial resources and caregiving experiences among dementia caregivers. Lastly, the study participants are spousal caregivers. Although this study highlighted the relationship between health risks and caregiving experience among spousal caregivers, adult child caregivers should be included in future studies to capture a full range of health status and behaviors among family caregivers for persons with ADRD.

Despite these limitations, the results of this study have numerous implications for researchers and clinical practitioners. The findings of this study contribute to healthcare providers’ recognition of health risks (i.e., sleep disturbance, fatigue, and less physical activity) among caregivers who take care of a spouse with ADRD. Our results can be used to develop indicators or algorithms to quantify caregiving intensity that can be integrated into the EHR. Identifying the intensity of caregiving experience within healthcare systems will enable systems to better assess health risks and tailor evidence-based interventions to meet the needs of dementia caregivers.

## Data Availability

The data that support the findings of this study are available on request from the corresponding author, J. Cho. The data are not publicly available due to the possibility that access to the data may compromise the privacy of research participants. Consequently, only those data that do not compromise patients’ privacy and associated documentation would be made available to users with appropriate Institutional Review Board approvals and a data-sharing agreement approved by the user’s institution and Baylor Scott and White Research Institute.
